# Supplementary material for: A novel art of continuous noninvasive blood pressure measurement
Source: Nat Commun. 2021 Mar 2;12:1387. doi: 10.1038/s41467-021-21271-8 (PMC7925606; doi:10.1038/s41467-021-21271-8)
Supplement: Supplementary file 1 — Supplementary Information [file 41467_2021_21271_MOESM1_ESM.docx]

## Supplementary Information for: Fortin et.al.

## A novel art of continuous noninvasive blood pressure measurement

# Supplementary Method 1

## Pseudocode of the filter cascade:

fc = [fc_1_, fc_2_, …, fc_N_] // Vector of cut-off frequencies from max to min

DSW = [DSW_1_, DSW_2_, …, DSW_N_] // Vector of “Down Sampling Window sizes”

UC = [UC_1_, UC_2_, …, UC_N_| // Vector of update coefficients (IIR-parameters)

// whereas $UC[n] = 1-e^{-2\pi\frac{fc[n]}{{fs}/{\prod_{1}^{n} DSW[n]}}}$

N: Number of cascaded filter stages – here 6

N_rhythm_: Index to cascade stage with fc[N_rhythm_] for v_Rhythm_(t) – here 3

vSum [N]: Vector of summed v(t)

vN [N]: Vector of down sampling counters

vFilt[n]: Vector of filtered signals

**CascadeFilter** (int n, float v, int N)

vSum[n] = vSum]n] + v

vN[n] = vN[n| + 1

if (vN[n] == DSW[n])

vFilt[n] = vFilt[n] + (vSum[n]/vN[n] – vFilt[n])*UC[n] // *****

vSum[n] = vN[n] = 0

if (n<N)

**CascadeFilter** (n+1, vFilt[n], N)

return

***** NOTE: vFilt[n] on the right side of the equation contains the old (n-1) value and is then overwritten with the new value on the left side of the equation

**NewData** (float v) // New sample v has been created

// called every 4ms -> 250Hz sampling frequency

**CascadeFilter** (0, v, N)

v_Pulse_ = v – vFilt[N_rhythm_]

v_VCT_ = v – vFilt[N]

v_Rhythm_ = vFilt[N_rhythm_] – vFilt[N]

v_dRhythm_ = vFilt[N_rhythm_+1] – vFilt[N_rhythm_-1]

The cut off frequencies are set to: [0.05, 0.05, 0.05, 0.01, 0.01, 0.01]

Down Sampling Window sizes are set to: [5, 1, 1, 5, 1, 1]

resulting in sampling frequencies for cascade 0-2 of 50Hz and for cascade 3-5 of 10Hz.

## Supplementary Method 2

## Pseudocode of the basic antiresonance filter elements:

**AdaptiveNotch** (float x) // called every 4ms -> 250Hz sampling frequency

// Downsampling from f_s_=250Hz to f_ds_=5Hz -> DSW = 50

x_ds_Sum += x; DS_count_++;

if (DS_count_ == DSW)

x_ds_[i] = x_ds_Sum / DS_count_ X_ds_Sum = DS_count_ = 0

// Select the total frequency area, where resonance can occur:

x_bp_[i] = FIR-Butterworth Band-pass-Filter

// A high power indicates possible resonance

Pow[i] = UC_Pow_ * x_bp_[i]^2 + (1-UC_Pow_) * Pow[i-1]

// FIR Notch Filter with estimated resonance

y_fir_[i] = x_bp_[i] - 2*λ[i]*X_bp_[i-1] + x_bp_[i-2]

// Adapt the resonance frequency indicator λ with Steepest Descent

if (Pow[i] > Threshold)

λ[i] = λ[i-1] + µ/2* Y_fir_[i]* x_bp_[i-1]

// Calculate periodic length T_r_ of the resonance

T_r_[i] = 2*π / (f_ds_ * arccos(λ[i]))

// Calculate adaptive gain factor α

α[i] = A_norm_^(A_exp_/T_r_[i])

// Calculate IIR-coefficients A_iir_ & B_iir_

B_iir_[i] = {1 -2λ[i] 1} * (1+α[i])/2

A_iir_[i] = {1 -λ[i]*(1+α[i]) α[i]}

y_iir_[i] = IIR-Notch with B_iir_[i] / A_iir_[i]

return x - x_ds_[i] + y_iir_[i] // Replace average with filtered signal

## Supplementary Method 3

## Pseudocode for beat detection:

bool **BeatDetected** (float v) // called every 4ms -> 250Hz sampling frequency

- each state-of-the-art beat detector for pulse detection can be used
- we use a modified version of the beat detector concept described in Supplementary Reference 1.
- This open-source algorithm was adapted to the nature of an inverted PPG-signal

returns TRUE, if a beat has been detected, otherwise FALSE

## Supplementary Method 4

## Pseudocode for the open loop phase:

The software algorithm for the open loop phase processes the following steps:

1. Continuously measure v(t) as well as p_c_(t) at a sampling frequency (fs) of 250 Hz.
2. Apply different contact pressures p_c_(t) to the finger. Although a pressure ramp would be suitable, we use the pressure steps implemented in the CNAP HD software.
3. Send v(t) to the filter cascade and afterwards to the beat detector.
4. Measure the v_Pulse_(t) amplitude after each detected beat and remember those amplitudes as well as their corresponding p_c_(t).
5. At the maximum amplitude, take the corresponding p_c_(t) as the starting mBP and remember this value as P_0_.
6. Calculate coefficients *c_BI_*, *c_P_*, *c_D_* and *c_I_* for VCT by using the maximum v_Pulse_. As v(t) is a dimensionless digital signal, these coefficients will highly depend on the PPG-system and its amplifiers.
7. Sets control variables y_I_, V_n_, PI, sumV_n_ to zero and P_n_ to P_0_.

## Supplementary Method 5

## Pseudocode of the main control function:

**VCT_Controller** (float v) // called every 4ms -> 250Hz sampling frequency

**NewData** (v) // Call to the filter cascades and create

// v_Pulse_, v_VCT_, v_Rhythm_ and v_dRhythm_

// Continuously tracking the physiological rhythms

$y_{PD}=c_{P}\cdot v_{Rhythm}+ c_{D}\cdot v_{dRhythm}$ // proportional and differential part

$y_{I}+=c_{I}\cdot y_{PD}$ // the summation builds the integral part

// Reconstruct very slow changing BP changes

V_n_ += v_VCT_  // Builds the beat-to-beat integral controller

PI += 4ms

if **BeatDetected** (v_Pulse_)

V_n_ = V_n_ / PI

sumV_n_ += V_n_

P_n_ = P_0_ - *c_BI_* * sumV_n_ - *c_BP_* * V_n_

V_n_ = PI = 0

// Calculate contact pressure

$p_{C}\left( t \right)=P_{n}-y_{I}-y_{PD}$

// Call antiresonance

p_c_(t) = **AdaptiveNotch** (p_c_(t))

Apply p_c_(t) to the finger

Supplementary Reference 1

Zong, W., Heldt, T., Moody, G. B., & Mark, R. G. An open-source algorithm to detect onset of arterial blood pressure pulses. *Computers in Cardiology* **30**, 259–262 (2003).
